# Venus or Æsculapius?

**Published:** 1888-06-30

**Authors:** 


### VENUS OR ÆSCULAPIUS?

You ask me, maiden, whether love still lives:
He may, perhaps, but not a sign he gives;
"'Tis sign of love to sigh,"
You say, "Not so;" say I,
'Tis indigestion!

When eyes grow dim, and rosy cheeks turn pale,
When appetite and faith in feeding fail,
You smile and say "'Tis love!"
Alas! ye gods above,
'Tis only liver!

Or where a maiden's colour comes and goes,
In blushes scarlet as a damask rose;
"She loves," I hear you say,
My friend, but lack-a-day,
'Tis mostly temper!

If she puts on becoming frocks to please
A friend, and then proceeds that friend to tease,
You cry, "'Tis Cupid's *rôle*;"
But really, 'pon my soul,
She's self conceited!

Absence of mind and lack of peaceful sleep,
A constant falling into dreamings deep,
You think a lover's way;
But all the doctors say,
Dyspepsia's!

If tears suffuse the eyes and wet the cheek,
The reason's plain, and is not far to seek:
No sign of love is this;
Methinks it surely is
But Hypochondria!

A lover's wild unless his lady smiles—
To see her home he'll walk at least five miles;
You think 'tis Love's dread dart!—
Bless your romantic heart,
He's got the fidgets!

